# The aged hematopoietic system promotes hippocampal‐dependent cognitive decline

**DOI:** 10.1111/acel.13192

**Published:** 2020-07-21

**Authors:** Lucas K. Smith, Evgenia Verovskaya, Gregor Bieri, Alana M. Horowitz, Saskia N. I. von Ungern‐Sternberg, Karin Lin, Peter Seizer, Emmanuelle Passegué, Saul A. Villeda

**Affiliations:** ^1^ Department of Anatomy University of California San Francisco San Francisco CA USA; ^2^ Biomedical Sciences Graduate Program University of California San Francisco San Francisco CA USA; ^3^ The Eli and Edyth Broad Center for Regenerative Medicine and Stem Cell Research San Francisco CA USA; ^4^ Columbia Stem Cell Initiative Department of Genetics and Development Columbia University Irving Medical Center New York NY USA; ^5^ Department of Cardiology and Cardiovascular Medicine University of Tübingen Tübingen Germany; ^6^ Neuroscience Graduate Program University of California San Francisco San Francisco CA USA

**Keywords:** aging, cognition, cyclophilin A, hematopoietic system, hippocampus

## Abstract

The aged systemic milieu promotes cellular and cognitive impairments in the hippocampus. Here, we report that aging of the hematopoietic system directly contributes to the pro‐aging effects of old blood on cognition. Using a heterochronic hematopoietic stem cell (HSC) transplantation model (in which the blood of young mice is reconstituted with old HSCs), we find that exposure to an old hematopoietic system inhibits hippocampal neurogenesis, decreases synaptic marker expression, and impairs cognition. We identify a number of factors elevated in the blood of young mice reconstituted with old HSCs, of which cyclophilin A (CyPA) acts as a pro‐aging factor. Increased systemic levels of CyPA impair cognition in young mice, while inhibition of CyPA in aged mice improves cognition. Together, these data identify age‐related changes in the hematopoietic system as drivers of hippocampal aging.

## Graphical abstract

The aged systemic milieu promotes cellular and cognitive impairments in the hippocampus. Here, we report that heterochronic hematopoietic stem cell (HSC) transplantation impairs hippocampal neurogenesis, decreases synaptic marker expression, and elicits age‐related cognitive impairments in young mice. We identify a number of factors that are elevated in the blood of young mice reconstituted with old HSCs, of which cyclophilin A (CyPA) acts as a pro‐aging factor. Increased systemic levels of CyPA impair cognition in young mice, while inhibition of CyPA in aged mice improves cognition.

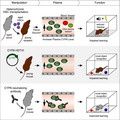

## 1 INTRODUCTION, RESULTS, DISCUSSION

Exposure to the aging systemic milieu, through models such as heterochronic parabiosis, promotes cellular, molecular, and structural changes in the brain that lead to cognitive decline (Katsimpardi et al., 2014; Smith et al., 2015; Villeda et al., 2011; Yousef et al., 2019). To date, studies have focused on the role of circulating factors, such as CCL11, B2M, and VCAM‐1, as mediators of the pro‐aging effects of old blood on the brain (Das et al., 2019; Smith et al., 2015; Villeda et al., 2011; Yousef et al., 2019). Despite the fact that the pro‐aging factors identified to date are immune‐related molecules (Smith, White, & Villeda, 2018), whether the aging hematopoietic system promotes hippocampal aging has not yet been investigated.

**Figure 1:**
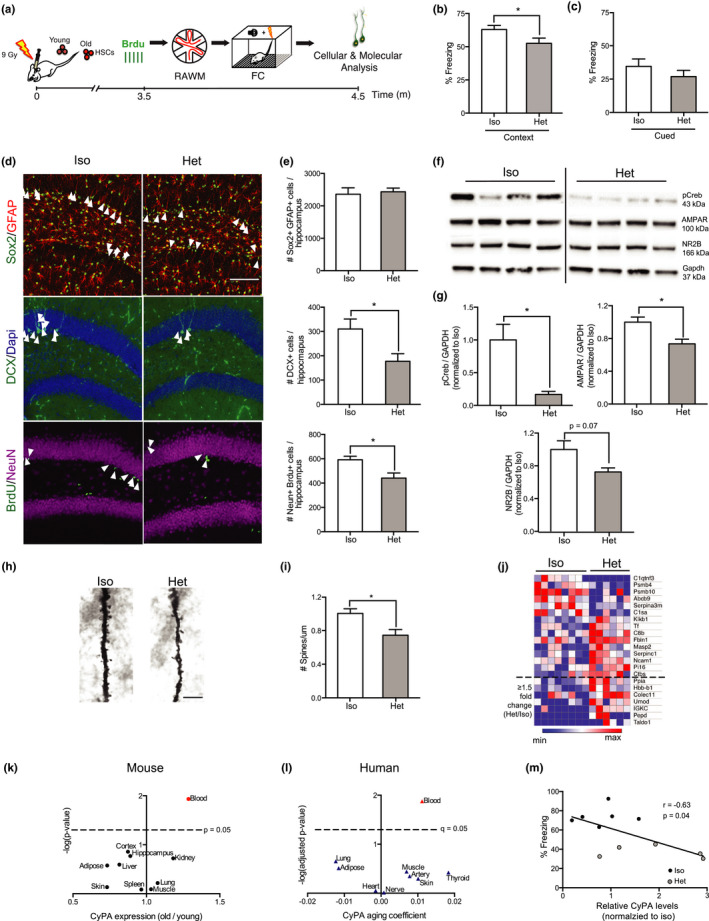
The aged hematopoietic system promotes cellular and cognitive hallmarks of hippocampal aging. (a) Schematic showing isochronic (Iso) and heterochronic (Het) HSC transplantation paradigm in young mice. (b, c) Hippocampal‐ and amygdala‐dependent memory was assessed in Iso and Het mice by contextual (b) or cued (c) fear conditioning (FC), respectively (*n* = 17‐19/group). (d, e) Representative fields (d; scale bar = 100 μm) and quantification (e) of Sox2+/GFAP+ (*n* = 5‐8/group), DCX+ (*n* = 7‐8/group), and NeuN+/BrdU+ (*n* = 10‐13/group) cells in Iso and Het HSC‐reconstituted mice. (f, g) Representative Western blot (f) and quantification (g) of hippocampal lysates from Iso and Het mice, probed with anti‐pCreb, AMPAR, NR2B, and GAPDH (*n* = 4‐5/group). (h, i) Representative Golgi stain (h; scale bar = 5 μm) and quantification (i) of dendritic spine density on tertiary branches (*n* = 4‐5/group). (j) Heat map of proteins differentially expressed (*p* < 0.05) between plasma of Iso and Het HSC‐reconstituted young mice, as determined by label‐free mass spectrometry (*n* = 6‐8/group). (k) CyPA expression across mouse tissues during aging by qPCR (*n* = 5–6/group). (l) Aging coefficients for CyPA across human tissues from RNA‐seq dataset (*n* = 83–156 subjects/tissue). (m) Correlation of plasma CyPA levels assessed by Western blot analysis, with percent time freezing in contextual FC in Iso and Het HSC‐reconstituted young mice (*n* = 5–6/group). All data shown as the mean + *SEM*. *p < 0.05. *t* test (b, c, e, g, i–k). Pearson's correlation (m).

In mice and humans, the hematopoietic system undergoes many functional and structural changes during aging, characterized by myeloid expansion, decreased immunity, and chronic low‐grade inflammation (Van Zant & Liang, 2012). We hypothesized that these cellular changes contribute to hippocampal aging through the accumulation of pro‐aging immune factors in old blood. Many of the age‐related changes observed in old blood have roots in hematopoietic stem cell (HSC) aging (Kim, Moon, & Spangrude, 2003; Pang et al., 2011). Therefore, we employed a heterochronic HSC transplantation model to test how exposure to an aged hematopoietic system contributes to hippocampal aging (Figure 1a). Young (2 months) recipient mice were sublethally irradiated (9 Gy) and transplanted with HSCs isolated from young (2 months) or old (24 months) donors, generating isochronic (Iso) and heterochronic (Het) HSC‐reconstituted young mice. Blood chimerism was assessed by measuring the proportion of CD45.2 donor cells in CD45.1 recipient mouse blood by flow cytometry (Figure S1a,b). Blood derived from old HSCs exhibited characteristic age‐related myeloid bias 4.5 months post‐transplantation (Figure S1c,d). Animals showed no signs of illness or weight loss regardless of treatment (Figure S1e).

To investigate whether exposure to an old hematopoietic system elicits age‐related cognitive impairments, we assessed hippocampal‐dependent associative fear memory and spatial memory using contextual fear conditioning (FC) and radial arm water maze (RAWM) behavioral paradigms, respectively. During contextual FC, Het HSC‐reconstituted young mice exhibited decreased freezing during testing compared with Iso controls (Figure 1b), indicating impaired hippocampal‐dependent memory. No differences were observed in baseline freezing during the training portion of the task (Figure S1f), or in the amygdala‐dependent cued FC paradigm (Figure 1c). During RAWM, all mice showed similar swim speeds (Figure S1g) and learning capacity during the training portion of the task (Figure S1h). Moreover, exposure to an aged hematopoietic system did not result in robust impairments in spatial memory during the testing portion of the task (Figure S1h). These data indicate that the aged hematopoietic system drives impairments in hippocampal‐dependent associative fear memory.

We next sought to assess cellular changes in the hippocampi of Het HSC‐reconstituted young mice that might contribute to cognitive decline. Hippocampal neurogenesis has been shown to decline with aging and after exposure to an aged systemic milieu (Villeda et al., 2011). Therefore, we tested whether exposure to an old hematopoietic system impairs neurogenesis by immunohistochemical analysis 4.5 months post‐HSC transplantation (Figure 1d,e). Although no difference was observed between groups in the number of Sox2+/GFAP+neural stem cells in the dentate gyrus (DG), young mice exposed to an old hematopoietic system had decreased numbers of doublecortin (DCX)‐positive immature neurons compared with controls (Figure 1d,e). To assess neuronal differentiation and survival, we employed a long‐term bromodeoxyuridine (BrdU) incorporation paradigm, in which mature neurons derived from proliferating stem cells retain BrdU labeling (Figure 1a). We observed decreased numbers of BrdU+cells co‐labeled with the mature neuronal marker NeuN in the DG of Het HSC‐reconstituted young mice compared with Iso controls (Figure 1d,e). These data indicate that exposure to an old hematopoietic system impairs hippocampal neurogenesis.

We next investigated whether exposure to an aged hematopoietic system elicits synaptic changes in the hippocampus at a molecular and structural level. Activation of the transcription factor, CREB, via phosphorylation, has been implicated in age‐related cognitive decline and rejuvenation (Villeda et al., 2014; Yu, Curlik, Oh, Yin, & Disterhoft, 2017). Correspondingly, we assessed the levels of CREB phosphorylation (pCreb) and observed a decrease in Het HSC‐reconstituted young mice compared with Iso controls (Figure 1f,g). Moreover, we observed decreased expression of the synaptic markers AMPAR and the NMDA receptor subunit NR2B (Figure 1f,g), both of which have previously been shown to decline with hippocampal aging (Shi et al., 2007; Wheatley et al., 2019). At a structural level, we examined dendritic spine density in granule cell neurons and observed a decrease in Het HSC‐reconstituted young mice (Figure 1h,i). Together, these data indicate that the old hematopoietic system leads to an age‐related decrease in synaptic density in the hippocampus.

To gain mechanistic insight into how the old hematopoietic system exerts its deleterious effects on cognition, we assessed peripheral immune cell infiltration into the hippocampus. Immunohistochemical identification of CD45.2+ hematopoietic cells in the DG of CD45.1 recipient mice revealed low and equivalent levels of immune cell infiltration in Het and Iso HSC‐reconstituted young mice (Figure S2a,b). While we cannot exclude the possible contribution of these small numbers of peripheral immune cells, we hypothesized that the pro‐aging effects of the old hematopoietic system are predominantly mediated through peripheral changes in circulating blood factors.

We performed unbiased proteomic analysis on blood plasma collected from Het and Iso HSC‐reconstituted young mice 4.5 months post‐transplantation. Using label‐free mass spectrometry, we identified 22 factors that were differentially expressed between Het and Iso HSC‐reconstituted young mice (Figure 1j). To identify potential pro‐aging factors, we focused analysis on proteins that were upregulated ≥1.5‐fold in the plasma of Het HSC‐reconstituted young mice (dotted line, Figure 1j). Of these, the most significantly upregulated cytokine was cyclophilin A (CyPA, encoded by *Ppia*)—an intracellular protein‐containing peptidyl‐prolyl cis‐trans isomerase activity that is secreted in response to inflammatory stimuli (Nigro, Pompilio, & Capogrossi, 2013). Elevated CyPA plasma levels were confirmed in Het HSC‐reconstituted mice by Western blot analysis (Figure S3a,b). In an independent cohort of naïve mice, we detected an age‐related increase in CyPA expression in blood cells by qPCR (Figure 1k). No age‐related changes in CyPA expression were observed in other tissues (Figure 1k). Examination of a previously published human RNA‐seq dataset from the GTEx project (Yang et al., 2015) demonstrated a similar increase in CyPA in human blood cells during aging (Figure 1l). We next analyzed the relationship between CyPA plasma levels and contextual FC performance in Iso and Het HSC‐reconstituted mice. We found an inverse correlation between CyPA levels and cognitive performance (Figure 1m) positing CyPA as a potential pro‐aging factor with relevance to cognition.

**Figure 2:**
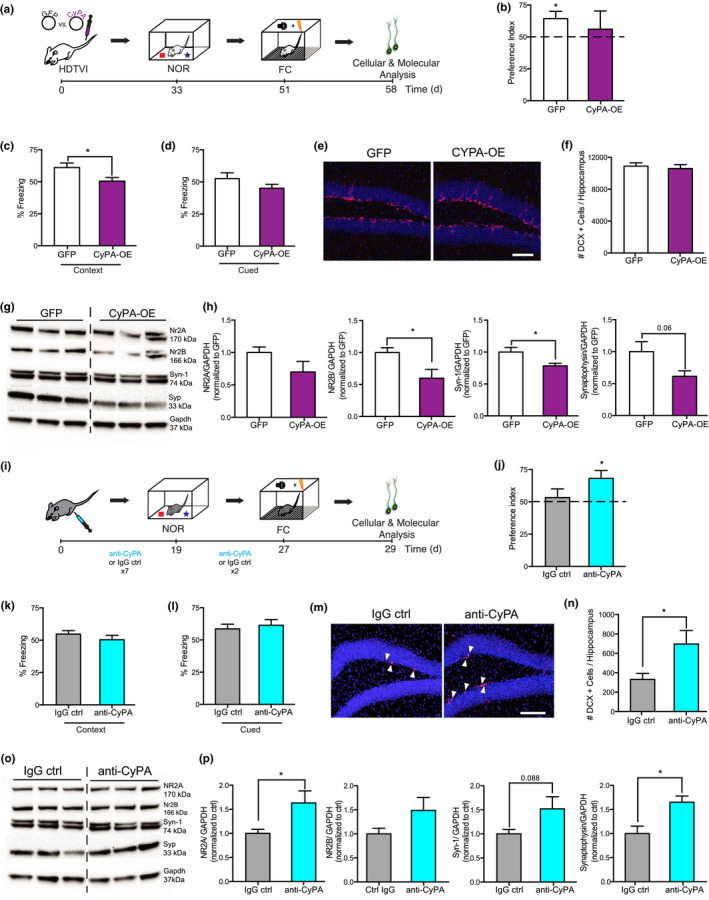
Cyclophilin A regulates cellular and cognitive hallmarks of hippocampal aging. (a) Schematic showing hydrodynamic tail vein injection (HDTVI)‐mediated CyPA overexpression (OE) paradigm in young mice. (b–d) Young CyPA‐OE or GFP control mice were tested in novel object recognition (b; NOR; *n* = 8–12/group), contextual fear conditioning (c; FC; *n* = 15–16), and cued FC (d; *n* = 15–16). (e, f) Representative fields (e; scale bar = 100 μm) and quantification (f) of DCX+ cells (*n* = 11/group), in DG of young CyPA‐OE and GFP control mice. (g, h) Representative Western blot (g) and quantification (h) of hippocampal lysates from young CyPA‐OE and GFP control mice, probed with anti‐NR2B, synapsin‐1 (Syn‐1), synaptophysin (Syp), and GAPDH (*n* = 5/group). (i) Schematic showing CyPA inhibition paradigm in aged mice. (j–l) Aged mice treated with an anti‐CyPA antibody or IgG isotype control (ctrl) were tested in NOR (j; *n* = 10–14/group), contextual FC (k; *n* = 10–14/group), and cued FC (l; *n* = 10–14/group). (m, n) Representative fields (m; scale bar = 100 μm) and quantification (n) of DCX+ (*n* = 8–10/group), in anti‐CyPA or IgG ctrl‐treated old mice. (o, p) Representative Western blot (o) and quantification (p) of hippocampal lysates from anti‐CyPA or IgG ctrl‐treated old mice, probed with anti NR2A, Syn‐1, Syp, and GAPDH (*n* = 5/group). All data shown as the mean + *SEM*. *p < 0.05. *t* test (c, d, f, h, k, l, n, p). One‐sample *t* test vs. hypothetical mean of 50 (b, j).

To test whether increasing systemic CyPA levels are sufficient to elicit age‐related cognitive or cellular impairments, we utilized a hydrodynamic tail vein injection (HDTVI) in vivo transfection approach, wherein young (2 months) mice were intravenously injected with overexpression constructs encoding either CyPA or GFP control (Figure 2a). The HDTVI‐mediated increase in plasma CyPA levels was confirmed using a HiBiT‐tagged CyPA and luminescent detection approach (Figure S3c). Animals showed no signs of illness or weight loss regardless of treatment (Figure S4a). One month after HDTVI, we assessed hippocampal‐dependent object recognition and associative fear memory using novel object recognition (NOR) and contextual FC behavioral paradigms, respectively (Figure 2b,c). In the testing portion of the NOR task, young control mice exhibited a significant preference for the novel object over the familiar object, consistent with proper cognitive function; however, this preference was lost in young mice overexpressing CyPA (Figure 2b). In contextual FC, we observed decreased freezing in young mice overexpressing CyPA mice compared with controls (Figure 2c). No differences were observed in baseline freezing (Figure S4b) or in cued FC (Figure 2d). At the cellular level, we examined hippocampal neurogenesis and observed no differences in the number of DCX‐positive immature neurons between treatment groups (Figure 2e,f). At the molecular level, we assessed the expression of synaptic markers in the hippocampus. Increased systemic CyPA resulted in lower levels of NMDA receptor subunit NR2B and the presynaptic markers synapsin‐1 and synaptophysin compared with control conditions (Figure 2g,h). These data indicate that increasing systemic CyPA promotes age‐related cognitive decline and decreased levels of key proteins important for synapse function.

Last, we explored whether inhibition of systemic CyPA in aged animals could ameliorate cognitive and cellular hallmarks of hippocampal aging. Aged (19 months) mice were administered with an anti‐CyPA neutralizing antibody (von Ungern‐Sternberg et al., 2017) or IgG isotype control, 9 times over 1 month, via intraperitoneal injection (Figure 2i). No differences in weight were observed between groups (Figure S4c). Hippocampal‐dependent cognition was examined by NOR and contextual FC behavioral paradigms (Figure 2j,k). In the testing portion of the NOR task, aged control mice failed to differentiate the novel from familiar object (Figure 2j), consistent with impaired cognitive function. However, aged mice treated with an anti‐CyPA antibody exhibited a significant preference for the novel object (Figure 2j), indicating improved hippocampal‐dependent memory. No differences in freezing were observed in FC (Figure 2k,l; Figure S4d). At the cellular and molecular levels, inhibition of systemic CyPA in aged mice increased the number of DCX‐positive immature neurons (Figure 2m,n) and increased expression of the NMDA receptor subunit NR2A and the presynaptic markers synapsin‐1 and synaptophysin (Figure 2o,p). These data indicate that targeting extracellular CyPA at old age is sufficient to improve object recognition memory, promote neurogenesis, and increase the levels of key proteins important for synapse function.

Cumulatively, our data demonstrate that age‐related changes in the hematopoietic system promote molecular, cellular, and cognitive hallmarks of hippocampal aging. We identify CyPA as a pro‐aging factor whose expression is elevated in the blood of het HSC‐reconstituted young mice. While the cellular source of CyPA in het HSC‐reconstituted young mice remains to be elucidated, increasing CyPA in the blood of young mice, by HDTVI‐mediated overexpression of CyPA, impaired hippocampal‐dependent cognition. Moreover, we found that the systemic inhibition of CyPA improved cognition in aged mice. Notably, inhibiting CyPA has been demonstrated to be neuroprotective in a mouse model of amyotrophic lateral sclerosis (Pasetto et al., 2017). In humans, elevated cerebrospinal fluid CyPA levels have recently been associated with cognitive impairments in Alzheimer's disease patients expressing apolipoprotein E4 (Montagne et al., 2020). Moreover, in humans elevated CyPA plasma levels accompany a number of inflammatory age‐related diseases, including diabetes (Ramachandran et al., 2014), and cardiovascular disease (Ohtsuki et al., 2017; Satoh et al., 2013). In these studies, CyPA plasma levels were also found to be elevated with aging (Ohtsuki et al., 2017; Ramachandran et al., 2014; Satoh et al., 2013). While little is known about the role of CyPA in aging, recent proteomic analysis using mass spectrometry has identified CyPA as part of the senescence‐associated secretory phenotype (SASP) (Wiley et al., 2019). Ultimately, our data identify the aged hematopoietic system, and downstream circulating immune factors, as potential therapeutic targets to restore cognitive function in the elderly.

## 2 MATERIALS AND METHODS

### 2.1 Mice

Young (2 months) and old (19–24 months) wild‐type C57BL/6J and B6.SJL/BoyJ mice were obtained from the Jackson Laboratory and subsequently maintained in‐house under specific pathogen‐free conditions under a 12‐hr light–dark cycle. All studies were done in male mice. The numbers of mice used to result in statistically significant differences were calculated using standard power calculations with *α* = 0.05 and a power of 0.8. We used an online tool (http://www.stat.uiowa.edu/~rlenth/Power/index.html) to calculate power and sample size on the basis of experience with the respective tests, variability of the assays, and interindividual differences within groups. All animal handling and use were in accordance with institutional guidelines approved by the University of California, San Francisco (UCSF) Institutional Animal Care and Use Committee (IACUC).

### 2.2 Irradiation and HSC transplantation

For transplantation studies, 2‐month‐old CD45.1 C57Bl/6‐Boy/J recipient mice were sublethally irradiated (9 Gy, administered in split doses, 3 hr apart) using a ^137^Cs source (J.L. Shepherd). 2000 purified CD45.2 C57BL/6J HSCs were transplanted by retro‐orbital injection to recipient mice, within 24 hr of irradiation. Recipient mice were maintained on antibiotic‐containing water for 4 weeks post‐transplantation. Blood chimerism was assessed every 5 weeks by retro‐orbital bleeding and flow cytometry. Mice with <80% blood chimerism, 3 months post‐transplantation, were excluded from further analysis.

### 2.3 Flow cytometry

HSCs were isolated as previously described (Ho et al., 2017). Bone marrow was obtained from leg, arm, and pelvic bones, isolated in Hanks' buffered saline solution (HBSS) supplemented with 2% heat‐inactivated FBS. Erythrocytes were lysed in ACK (150 mM NH4Cl/10 mM kHCO3/10 mM EDTA). Bone marrow cells were further purified using a Ficoll gradient (Histopaque 119, Sigma‐Aldrich). c‐Kit‐positive cells were enriched for with c‐Kit microbeads (Miltenyi Biotec) and MACS separation LS columns (Miltenyi Biotec). Bone marrow cells were then incubated with purified rat anti‐mouse antibodies for CD3 (BioLegend), B220 (eBioscience), CD4 (eBioscience), CD5 (eBioscience), CD8 (eBioscience), CD11b (eBioscience), GR‐1 (eBioscience), Ter119 (eBioscience), followed by goat anti‐rat PE‐Cy5 (Invitrogen). Cells were blocked with purified rat IgG (Sigma‐Aldrich) and stained with c‐Kit‐APC‐eFluor780 (eBioscience), Sca‐1‐PB (BioLegend), CD48‐A647 (BioLegend), CD150‐PE (BioLegend), and Flk2‐bio (eBioscience), followed by SA‐PE‐Cy7 (eBioscience). Stained bone marrow cells were resuspended in 1 μg/ml propidium iodide (PI) to exclude dead cells. HSCs were isolated by double sorting with a FACSAria II (BD Biosciences). For blood chimerism analysis, erythrocytes were lysed in ACK, and cells were maintained in HBSS +2% heat‐inactivated FBS. Blood was stained with CD11b‐PE‐Cy7 (eBioscience), Gr1‐PB (eBioscience), B220 APC‐Cy7 (eBioscience), CD3 APC (eBioscience), and TER119‐PE‐Cy5 (eBioscience), CD45.1‐PE (eBioscience), and CD45.2‐FITC (eBioscience). Stained blood cells were resuspended in 1 μg/ml PI (Thermo Fisher) to exclude dead cells. Chimerism was analyzed with a FACS LSR II using DIVA software (BD Biosciences).

### 2.4 BrdU administration

For long‐term BrdU labeling studies, 50 mg/kg of BrdU (Sigma‐Aldrich) was injected intraperitoneally into mice daily for 5 days. Mice were euthanized 33 days after first BrdU injection.

### 2.5 Immunohistochemistry

Tissue processing and immunohistochemistry were performed on free‐floating sections according to standard published techniques (Smith et al., 2015). Mice were anesthetized with 87.5 mg/kg ketamine and 12.5 mg/kg xylazine and transcardially perfused with 1× phosphate‐buffered saline (PBS). Brains were removed and fixed in phosphate‐buffered 4% paraformaldehyde, pH 7.4, at 4°C for 48 hr before cryoprotection in 30% sucrose. Brains were sectioned coronally, at 40 μM with a cryomicrotome (Leica Camera, Inc.), and stored in cryoprotective medium. Primary antibodies used were as follows: goat anti‐DCX (1:7000; Santa Cruz), rat anti‐BrdU (1:1000; Abcam), mouse anti‐NeuN (1:1000; Millipore), goat anti‐Sox2 (1:200, Santa Cruz), rabbit anti‐GFAP (1:1000, Dako), and anti‐CD45.2‐Alexa Fluor 647 (BioLegend). After overnight incubation, unconjugated primary antibody staining was revealed using fluorescence‐conjugated secondary antibodies (Life Technologies). For BrdU labeling, brain sections were pre‐treated with 2 N HCl at 37°C for 30 min and washed three times with Tris‐buffered saline with Tween (TBST) before incubation with primary antibody. All cells were counted in the DG of every sixth coronal hemibrain section through the hippocampus and analyzed by confocal or epifluorescent microscopy using a Zeiss Axio Observer Z1 or Axio Imager M2 microscope, respectively. CD45.2 cells were imaged as a Z‐stack and counted in a standardized box centered on the DG and covered part of the hilus, granular cell layer, and molecular layer. CD45.2 counts were normalized to volume.

### 2.6 Golgi staining and analysis

Mice were anesthetized with 87.5 mg/kg ketamine and 12.5 mg/kg xylazine, and brains were removed without perfusion. Golgi staining was performed using FD Rapid GolgiStain Kit (FD Neurotechnologies, Inc.; PK401), per manufacturer's instructions. Hemibrains were rinsed in Milli‐Q water and immersed in the impregnation solution (FD Solutions A + B) for 2 weeks (replacing impregnation solution one time, 24 hr after immersion). Samples were transferred into FD Solution C and incubated for 1 week (replacing FD Solution C one time, 24 hr after immersion). 200‐μm sections were cut using a cryomicrotome (Leica Camera, Inc.), and sections were mounted on gelatin‐coated slides (FD NeuroTechnologies) in a drop of FD Solution C. After drying, slides were rinsed in Milli‐Q H2O 2× for 4 min. Slides were transferred to developing solution (FD Solution D + E) for 10 min. Slides were then rinsed 2× in Milli‐Q water, 4 min each, and dehydrated in 50%, 75%, 95% (1 × 4 min each), and 100% ETOH (4× 4 min). Slides were then incubated in xylene 3×, 4 min each, and coverslipped with Permount (Fisher). Tertiary dendritic spines on granule cell neurons were imaged at 63×. Spines were counted on 2‐3 tertiary dendrites/neuron and 5–11 neurons/mouse.

### 2.7 Western blot analysis

Mouse hippocampi were dissected after perfusion of animals, snap‐frozen, and lysed in RIPA lysis buffer (500 nM Tris, pH 7.4, 150 mM NaCl, 0.5% sodium deoxycholate, 1% NP‐40, 0.1% SDS, and complete protease inhibitors; Roche). For analysis of synaptic genes, in the Iso and Het HSC‐reconstituted cohort and GFP or CyPA overexpression cohort, mice with >55% contextual freezing in the control group and <55% contextual freezing in the treatment group were used. For analysis of plasma CyPA levels in Iso and Het mice, samples were depleted of the most abundant proteins using Proteome Purify 2 Mouse Serum Protein Immunodepletion Resin (R&D Systems) according to the manufacturer's protocol. For analysis of HiBiT‐tagged CyPA in plasma of HDTVI mice, equal volumes of blood plasma were used. Samples were mixed with 4× NuPAGE LDS loading buffer (Invitrogen) and loaded on a 4%‐12% SDS‐polyacrylamide gradient gel (Invitrogen) and subsequently transferred onto a nitrocellulose membrane. The blots were blocked in 5% milk in TBST and incubated with rabbit anti‐pCREB (1:1000; Cell Signaling), rabbit anti‐AMPAR (1:3000; Abcam), mouse anti‐synaptophysin (1:1000; Millipore), rabbit anti‐NR2B (1:2000; Millipore), rabbit anti‐NR2A (1:4000; Millipore), rabbit anti‐synapsin‐1 (1:5000; Abcam), mouse anti‐GAPDH (1:5000; Abcam), and rabbit anti‐CyPA (1:200; Enzo Life Sciences). Horseradish peroxidase‐conjugated secondary antibodies and enhanced chemiluminescence (ECL) kit (GE Healthcare) were used to detect protein signals. Membranes were imaged using a ChemiDoc system (Bio‐Rad) and quantified using ImageJ software (Version 1.52a). GAPDH bands were used for normalization for hippocampal lysates. Equal loading of plasma was confirmed using Ponceau S solution (Sigma‐Aldrich). HiBiT was detected using the Nano‐Glo HiBit Lytic Detection System (Promega), and luminescence was recorded with ChemiDoc (Bio‐Rad).

### 2.8 qPCR

Tissue was dissected, snap‐frozen, and total RNA was extracted using TRIzol reagent (Invitrogen). cDNA was subsequently synthesized and mixed using the High‐Capacity cDNA Reverse Transcription Kit (Thermo Fisher Scientific, Cat# 4374966) and mixed SYBR FAST mix (Kapa Biosystems), and our primers of interest. Quantitative RT‐PCR was carried out in a CFX384 Real Time System (Bio‐Rad). The following primers were used: GAPDH (F‐AGGTCGGTGTGAACGGATTTG; R‐TGTAGACCATGTAGTTGAGGTCA) and PPIA (F‐GGCAAATGCTGGACCAAAC; R‐CATTCCTGGACCCAAAACG).

### 2.9 Tissue expression of CyPA (human)

Yang et al. (2015) identified age‐related gene expression changes in human RNA‐seq data collected by the Genotype‐Tissue Expression (GTEx) project (Ardlie et al., 2015). They examined tissue expression changes in nine tissues: subcutaneous adipose, tibial artery, left ventricle heart, lung, skeletal muscle, tibial nerve, skin, thyroid, and whole blood (n/tissue = 83–156 subjects, ages 20–70 years). Using a linear regression model, Yang et al. generated an aging coefficient for each gene detected in each tissue. Aging coefficients that are significantly >0 after false discovery rate adjustments (*q* < 0.05) are upregulated with age. We examined how CyPA expression changes with age in their dataset by graphing the aging coefficient of CyPA against the –log10 adjusted *q*‐value.

#### 2.9.1 Plasmid generation

RNA was isolated from adult mouse spleen tissue using TRIzol reagent (Thermo Fisher Scientific) and PureLink™ RNA Mini Kit following the manufacturer's instructions. The RNA concentration was determined via NanoDrop, and RNA was reverse‐transcribed using the High‐Capacity cDNA Reverse Transcription Kit (Thermo Fisher Scientific) and oligodT primers (Promega). The following primers were used for PCR amplification of the *PPIA*‐coding sequences and partial 3′ and 5′ untranslated regions (UTRs) from cDNA: CACCATGGTCAACCCCACCGTGTT (forward primer); GGTAAAATGCCCGCAAGTCA (reverse primer). The *PPIA* PCR product was purified, cloned into the pENTR D‐TOPO vector (Thermo Fisher Scientific), and sequence‐verified using standard M13F, M13R primers. The coding sequence was further amplified and cloned into a bicistronic mammalian expression plasmid using the restriction sites NheI and EcoRI. A canonical Kozak sequence (GCCACC) was included in the forward primer. For the C‐terminal HiBiT‐tagged PPIA, the nucleotide sequence coding for the 11‐amino acid HiBiT tag was included in the reverse primer. The resulting bicistronic plasmid vectors expressed untagged or HiBiT‐tagged Ppia and an IRES eGFP reporter using a CMV promoter. An empty IRES eGFP construct, based on the same plasmid, was used as a control for in vivo experiments. All coding sequences of the plasmids were verified by Sanger sequencing. Endotoxin‐free plasmid kits were used for plasmid preparation prior to in vivo use.

#### 2.9.2 Hydrodynamic tail vein injection

The hydrodynamic tail vein injection protocol was adapted from a previously described protocol (Kovacsics & Raper, 2014). Endotoxin‐free plasmids were prepared using the Qiagen Maxi‐Prep Plus Kit (VWR). CyPA, CyPA‐HiBiT, or GFP plasmid DNA (50 μg) was suspended in 10% body weight saline and injected in the tail vein in 5–7 s in young mice. For detection of HiBiT‐tagged CyPA, blood was drawn 24 hr post‐HDTVI. Following a similar timeline as previous pro‐aging studies (Smith et al., 2015), behavioral testing was started 33 days post‐HDTVI.

#### 2.9.3 CyPA neutralizing antibody administration

The anti‐CyPA antibody, 8H7 (Von Ungern‐Sternberg et al., 2017), or a monoclonal IgG2A isotype control (R&D Systems) was intraperitoneally administered to aged (19 months) mice (20 μg/kg). Prior to behavioral testing, antibodies were administered 7 times every third day, with two additional injections administered between behavioral testing.

#### 2.9.4 Radial arm water maze

Spatial learning and memory were assessed using the radial arm water maze (RAWM) paradigm according to established protocol (Alamed, Wilcock, Diamond, Gordon, & Morgan, 2006). In this task, the location of the goal arm, which contains a platform, remains constant throughout the training and testing phase, while the start arm is changed during each trial. On day 1 during the training phase, mice are trained for 15 trails, with trials alternating between a visible and hidden platforms. On day 2 during the testing phase, mice are tested for 15 trials with a hidden platform. Entry into an incorrect arm is scored as an error, and errors are averaged over training blocks (three consecutive trials).

#### 2.9.5 Contextual fear conditioning

In this task, mice learned to associate the environmental context (fear‐conditioning chamber) with an aversive stimulus (mild foot shock; unconditioned stimulus, US) enabling testing for hippocampal‐dependent contextual fear conditioning. As contextual fear conditioning is hippocampal‐ and amygdala‐dependent, the mild foot shock was paired with a light and tone cue (conditioned stimulus, CS) in order to also assess amygdala‐dependent cued fear conditioning. Conditioned fear was displayed as freezing behavior. Specific training parameters are as follows: Tone duration is 30 s; level is 70 dB, 2 kHz; shock duration is 2 s; and intensity is 0.6 mA. This intensity is not painful and can easily be tolerated but will generate an unpleasant feeling. On day 1, each mouse was placed in a fear‐conditioning chamber and allowed to explore for 2 min before delivery of a 30‐s tone (70 dB) ending with a 2‐s foot shock (0.6 mA). Two minutes later, a second CS‐US pair was delivered. On day 2, each mouse was first placed in the fear‐conditioning chamber containing the same exact context, but with no CS or foot shock. Freezing was analyzed for 2 min. One hour later, the mice were placed in a new context containing a different odor, cleaning solution, floor texture, chamber walls, and shape. Animals were allowed to explore for 2 min before being re‐exposed to the CS. Freezing was analyzed for 30 s after re‐exposure to CS. Freezing was measured using a FreezeScan video tracking system and software (Clever Sys, Inc) or EthoVision XT 11.5 tracking software (Noldus) and a Ugo Basile FC system.

#### 2.9.6 Novel object recognition

The novel object recognition task was adapted from a previously described protocol (Dubal et al., 2015). During the habituation phase (day 1), mice could freely explore an empty arena for 10 min. During the training phase (day 2), mice were exposed to two identical objects (either two striped scintillation vials or two lego constructions). Mice were allowed to explore the objects for 5 min. Mice that did not explore the familiar objects for more than 3 s during the training phase were excluded from analysis. During testing (day 3), one familiar object was replaced with a novel object (vial replaced with lego or lego replaced with vial), and mice could explore for 5 min. Time spent exploring each object was quantified using the Smart Video Tracking Software (Panlab; Harvard Apparatus).

#### 2.9.7 Plasma collection

At time of euthanasia, mouse blood was collected via intracardial bleeds into EDTA‐coated tubes. Plasma was generated by centrifugation of freshly (<30 min) isolated blood at 1,000 g. Aliquots were stored at −80°C until use.

#### 2.9.8 Label‐free mass spectrometry

For proteomic analysis of old hematopoietic system associated factors, plasma was collected from young Iso and Het HSC‐reconstituted that had exhibited >60% or <50% freezing in contextual FC, respectively. Plasma was collected from 8 individual mice per condition for a total of 16 samples. Samples were depleted of the most abundant proteins using Proteome Purify 2 Mouse Serum Protein Immunodepletion Resin (R&D Systems) according to the manufacturer's protocol. Depleted samples were buffer exchanged into water on a Corning Spin X 5 kD molecular weight cut off spin column and quantified by Qubit fluorometer (Life Technologies). Plasma proteins (50 μg) were reduced with dithiothreitol, alkylated with iodoacetamide, and digested with trypsin (Promega). Individual digested samples were processed by solid‐phase extraction using an Empore C18 (3 M) plate under vacuum. Briefly, columns were activated (400 μl 95% acetonitrile/0.1% TFA X2) and equilibrated (400 μl 0.1% TFA X4). Next, acidified samples were loaded and columns were washed (400 μl 0.1% TFA X2). Finally, peptides were eluted (200 μl 70% acetonitrile/0.1% TFA X2) and lyophilized for downstream processing. For mass spectrometry analysis, 2 μg of protein per sample was analyzed by nano LC‐MS/MS with a Waters NanoACQUITY interfaced to a Thermo Fisher Fusion Lumos Mass Spectrometer. Peptides were loaded on a trapping column and eluted over a 75 μm × 50 cm analytical column (Thermo Fisher P/N ES‐803) at 300 nl/min using a 3‐hr reverse‐phase gradient. The mass spectrometer was operated in data‐dependent mode, with the Orbitrap operating at 60,000 FWHM and 15,000 FWHM for MS and MS/MS, respectively. The instrument was run with a 3‐s cycle for MS and MS/MS, and APD was enabled. The data were processed with MaxQuant (version 1.6.0.13; Max Planck Institute for Biochemistry; (Tyanova, Temu, & Cox, 2016), which incorporates the Andromeda search engine. Using this program, the MS data were recalibrated, protein/peptide identification was made using the Andromeda database search engine, the database search results were filtered at the 1% protein and peptide FDR, and protein levels were quantified. The resulting MaxQuant output was further processed using Perseus (V 1.6.0.7; Max Planck Institute for Biochemistry). Two samples exhibiting clotting changes as described in Geyer et al. (2016) were excluded from analysis. Differentially expressed genes were identified by comparing LFQ intensities for each protein detected in Heterochronic HSC‐reconstituted mice compared with isochronic HSC‐reconstituted controls.

#### 2.9.9 Data and statistical analysis

Mice were randomized prior to treatment. Researchers were blinded throughout histological, biochemical, and behavioral assessments. Groups were un‐blinded at the end of each experiment upon statistical analysis. Data are expressed as mean + *SEM*. The distribution of data in each set of experiments was tested for normality using the D'Agostino–Pearson omnibus test or Shapiro–Wilk test. No significant differences in variance between groups were detected using an *F* test. Statistical analysis was performed with Prism 6.0 software (GraphPad Software). Means between two groups were compared with two‐tailed, unpaired Student's *t* test. Comparisons of means from multiple groups with each other or against one control group were analyzed with one‐way ANOVA followed by appropriate post hoc tests (indicated in figure legends). The data that support the findings of this study are available from the corresponding author upon reasonable request.

## CONFLICT OF INTEREST

The authors declare that they have no competing financial interests.

## AUTHOR CONTRIBUTIONS

L.KS. and S.A.V. developed concept and designed experiments. L.K.S. collected and analyzed data. E.V. assisted with HSC transplantation studies and analysis. G.B. and A.M.H. assisted with CyPA‐OE studies. S.U‐S. and P.S. generated and provided the antibody for CyPA inhibition studies. K.L. assisted with cognitive analysis. E.P. provided reagents and conceptual advice. L.K.S. and S.A.V. wrote the manuscript. S.A.V. supervised all aspects of this project. All authors had the opportunity to discuss results and comment on the manuscript.

## Supporting information

Fig S1

Fig S1‐S4

Supplementary Material

## Data Availability

The data that support the findings of this study are available from the corresponding author upon reasonable request.
