# Supplementary figures and images for: The aged hematopoietic system promotes hippocampal‐dependent cognitive decline
Source: Aging Cell. 2020 Jul 21;19(8):e13192. doi: 10.1111/acel.13192 (PMC7431826; doi:10.1111/acel.13192)

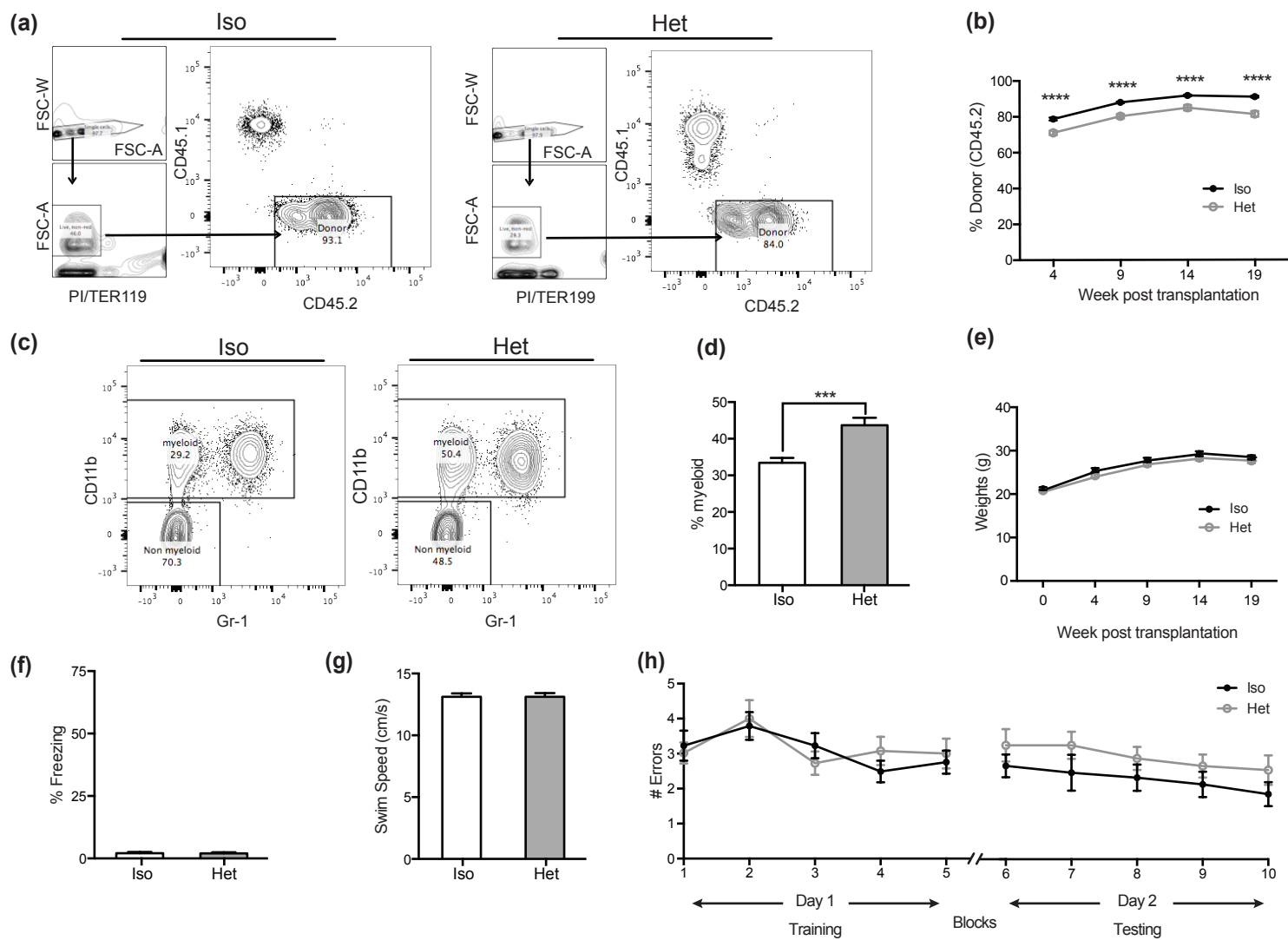

Figure S1

Supplement: Supplementary file 1 — Fig S1 [file ACEL-19-e13192-s002.pdf]

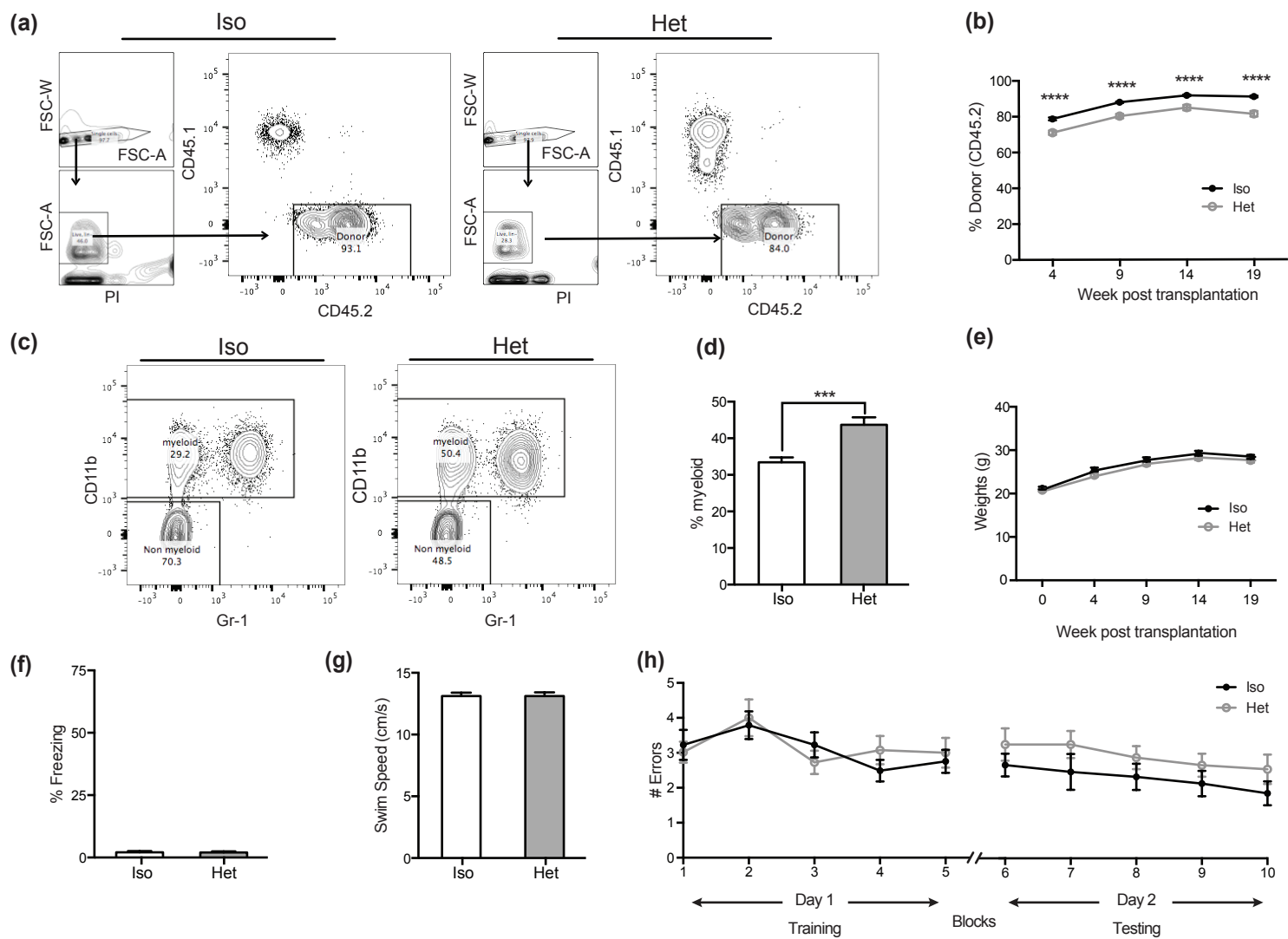

Figure S1

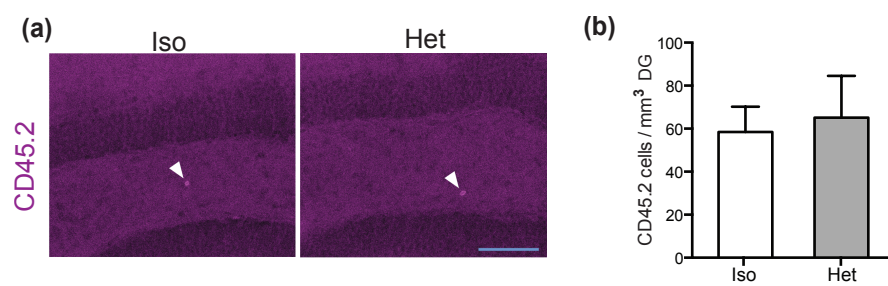

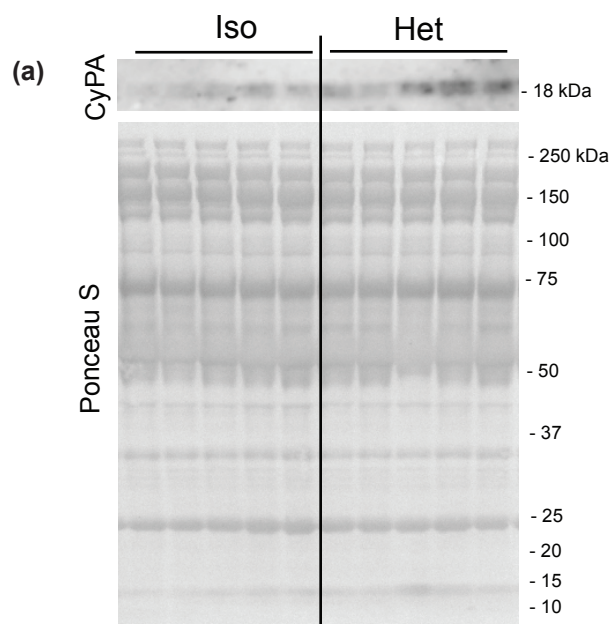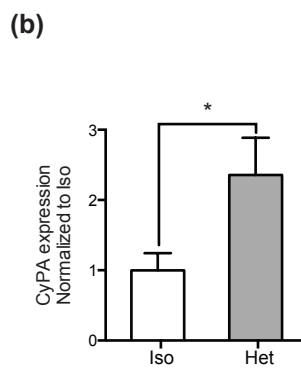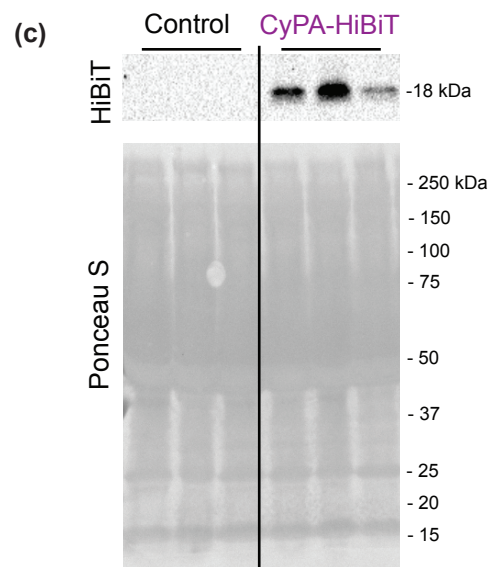

Figure S3

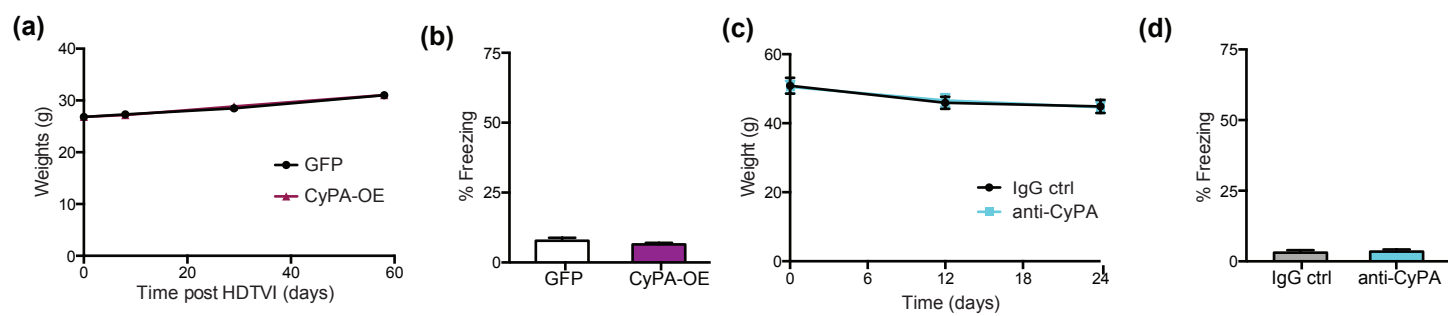

Figure S4

Supplement: Supplementary file 2 — Fig S1‐S4 [file ACEL-19-e13192-s001.pdf]
